# Might Vestibular “Noise” Cause Subclinical Balance Impairment and Falls?

**Published:** 2021-10-11

**Authors:** Andrew R Wagner, Ajit MW Chaudhari, Daniel M Merfeld

**Affiliations:** 1 School of Health and Rehabilitation Sciences, The Ohio State University, Columbus, Ohio, USA; 2 Otolaryngology-Head and Neck Surgery, Ohio State University Wexner Medical Center, Columbus, Ohio, USA; 3 Department of Biomedical Engineering, The Ohio State University, Columbus, Ohio, USA; 4 Department of Speech and Hearing Science, The Ohio State University, Columbus, Ohio, USA

**Keywords:** Vestibular, Noise, Imbalance, Falls, Aging, Sway

## Abstract

Falls are the leading causes of accidental injury in older adults and directly contribute to more than 600,000 deaths each year worldwide. Although the issue of falls is complex, balance dysfunction is one the principal contributors to the heightened incidence of falls in older adults. A nationally representative survey of older adults in the United States showed that an inability to stand on a foam pad with the eyes closed was associated with more than a six-fold increase in the odds of reporting “difficulty with falls.” As stability in the “eyes closed, on foam” condition is reliant upon intact vestibular cues, these data implicate age-related vestibular loss as a potential contributor to falls, yet, the specific causal mechanism explaining the link between age-related vestibular loss and imbalance/falls was not known. Here we review recent data showing that, vestibular perceptual thresholds, an assay of vestibular sensory noise, were found to, (1) account for nearly half of subclinical balance impairment in healthy older adults and (2) correlate with postural sway in healthy young adults. Based upon the identified links between balance dysfunction and vestibular noise in healthy adults, we posit the following causal chain: (a) increased “noise” in vestibular feedback - yielding a reduced signal-to-noise ratio in vestibular feedback-increases sway, (b) excessive sway leads to imbalance, and (c) imbalance contributes to falls. Identifying the “cause” of age-related balance dysfunction will inform the development of interventions tailored to prevent falls, and fall-related injuries, in the growing population of older adults.

## INTRODUCTION

A common consequence of aging is a progressive decline in balance [1–3] leading to a heightened risk for falls and fall-related injuries [4–6]. Imbalance can result from the decline in a number of different sensorimotor factors (e.g., strength [7], proprioception [8], vision [9]); however recent findings suggest that age-related vestibular loss may serve as one preeminent factor contributing to, and potentially causing, the observed incidence of age-related balance impairment and fall risk [10,11]. Identifying an element hypothesized to cause age-related imbalance poses a unique opportunity to intervene prior to the onset of falls and fall-related injuries, preserving the mobility and independence of the aging population.

Using a large nationally representative National Health and Nutrition Examination Survey (NHANES) sampling of older adults (N=5,086), Agrawal, et al. [12] found that the inability to stand with the eyes closed on a foam pad was associated with more than a six-fold increase in the odds of reporting a difficulty with falls in the past year (Odds Ratio=6.3, 95% CI 2.9 to 13.8). This test condition is often referred to as the “vestibular condition” of the modified Romberg Test of Balance as it is thought to require the subject to down weight the perturbed proprioceptive cues and absent visual cues in favor of the unperturbed vestibular cues [3]. As such, the finding by Agrawal was postulated to provide a link between age-related vestibular loss and falls. The strengthening of this correlation in the presence of concurrent dizziness (Odds ratio of 12.3, 95% CI 7.9 to 16.7) further bolstered this conclusion [12]. These results are robust because of the large, representative sample and the consideration of several potential confounders in the statistical analysis; however, the specific influence of vestibular loss on the human balance system cannot be inferred from performance on a balance task in isolation.

Balance represents one’s ability to maintain their center of mass within their base of support [3,13], allowing us to remain upright in the face of internal and external perturbations. Balance results from the contribution of several sensory systems, including but likely not limited to vision, proprioception, and vestibular [14–16]. Thus, while the “eyes closed, on foam” task likely prioritizes vestibular cues, it does so in concert with alternative sensorimotor inputs (e.g., proprioceptive, tactile), and thus cannot represent the specific effects of vestibular impairment [3]. Furthermore, just as balance assessments in isolation cannot allow us to fully account for the role that each sensory system plays in balance, these assessments also are unable to account for the multimodal nature of the human vestibular system and which of its subcomponents may be most important for balance [17].

The vestibular system senses both linear acceleration and gravity using two otolith organs and rotations using three orthogonally oriented semicircular canals. This complexity allows humans to sense rotations and translations of the head in six degrees of freedom and provides a suitable mechanism by which to sense, and subsequently respond to, balance perturbations. To identify the specific contributions of the vestibular system to age-related imbalance, the integrity of each of the five vestibular sensor pairs (i.e., vestibular organs are found in both inner ears) must be determined. Thus, while the ability to stand on foam with vision removed is suggestive of vestibular impairment, the relative effects of the specific vestibular end organs (semicircular canal or otolith) and associated sensory modalities (i.e., rotation, tilt, or translation sensation) on balance performance cannot be determined from balance testing in isolation. We posit that comparing specific, sensitive, quantitative measures of vestibular function to valid measures of balance performance can provide unique insights into the role of vestibular cues in postural sway.

## LITERATURE REVIEW

Bermúdez-Rey and colleagues measured a battery of vestibular perceptual thresholds, alongside the “eyes closed, on foam” balance task (identical to that used in the NHANES study) in a sample of 99 adults aged 18 to 89 [18]. These data showed large vestibular threshold increases above the age of 40. In a later multivariable analysis of this dataset, roll tilt perceptual thresholds were found to correlate with the likelihood of completing the balance task (p=0.006) [19]. Roll tilt describes the rotation of the head about a naso-occipital axis; as the rotation occurs about an axis aligned perpendicular to gravity, roll tilt activates the angular velocity sensing vertical semicircular canals as well as the tilt sensitive otolith organs. In a reanalysis of these data, Beylergil and colleagues showed that roll tilt thresholds accounted for (i.e., mediated) 46% of the effect of age on balance performance [20]. One possible explanation for these large correlations is that roll tilt represents an ecologically valid stimulus for the control of upright posture since the head is tilted away from gravitational vertical. However, given that vestibular thresholds assay vestibular noise, consistent with that explanation, we posit a more general supposition, that subclinical balance impairment increases as vestibular tilt noise increases [21]. While the categorical nature of the pass/fail, “eyes closed, on foam” balance test precludes a more sensitive analysis of the specific vestibular modalities (translation, rotation, tilt) relevant for the control of balance, we specifically note that increased roll tilt noise, consistent with increased roll tilt thresholds, would be expected to yield increased mediolateral sway.

The categorical test limitation was addressed in a recent publication by Karmali et al. in the Journal of Neurophysiology [22]. Karmali et al. compared a battery of thresholds, similar to what was used by Bermúdez-Rey, to postural sway quantified by measuring displacement of the center of pressure under various sensory conditions. They showed that in young to middle-aged populations (range 21-61 years), postural sway was selectively correlated with lateral translation thresholds and that the correlation was strengthened in conditions where proprioceptive cues were altered (eyes closed, on a sway referenced support). This finding suggests that the processing of head lateral translation cues may be a critical determinant for postural control in conditions where proprioceptive and visual cues are absent and/or degraded (e.g., walking across a plush carpet in the dark). In contrast to the findings of Bermúdez-Rey, roll tilt thresholds were not shown to correlate with these quantitative measures of postural sway. The age difference between the populations in these two studies may implicate roll tilt vestibular noise as having specific effects on age-related imbalance.

Here, we have described studies where vestibular thresholds have been compared to postural sway measured only under quiet stance conditions. Given the breadth of our definition of balance-which additionally includes dynamic anticipatory, and dynamic compensatory responses-quiet stance represents but one of several sub functions that comprise the human balance system. Nevertheless, we posit that the significant correlations observed between quiet stance postural sway and falls support the ability of quiet stance sway metrics to quantify the integrity of the human balance system. Thus, quiet stance sway metrics may provide a relatively simple screening test for fall risk.

Several investigators over the past 40 years have attempted to relate static, anticipatory, and compensatory balance measures to falls, with the static balance measures resulting in the most accurate predictions of falls. In the early 1990’s, Maki, et al. used quantitative measures of quiet stance postural sway to prospectively predict the incidence of falls over a 1-year follow-up period in a sample of 100 healthy adults [1]. They showed that falls, and in particular falls that occurred secondary to a perturbation to the center of mass or base of support, were best predicted (p=0.008) by mediolateral sway measured in an eyes closed quiet stance condition [1]. These findings appear consistent with the vestibular threshold findings reported above; recall that increased roll tilt thresholds represent noisier roll tilt sensation, and increased roll tilt noise should cause increased mediolateral sway.

In this study, Maki also tested dynamic compensatory and anticipatory postural sway as well as clinical measures; a stepwise analysis selected mediolateral sway during eyes closed quiet stance “as the single measure that best differentiated between fallers and nonfallers” [1]. Subsequent studies of larger populations by Thapa et al. (n=303) [23] and Stel et al. (n=439) confirmed the value of eyes closed quiet stance as a measure predictive of falls [24]. As previously mentioned, Agrawal reported a similar result even when quiet stance balance performance was quantified only as a categorical “pass/fail” metric [12]. In their sample of more than five-thousand older adults, passing or failing a quiet stance balance task was independently associated with fall risk even after controlling for age, sex, ethnicity, and cardiovascular risk factors [12]. These four independent sets of data clearly oppose the “common sense” supposition that balance tasks that reflect “real-world” challenges that typically yield falls (e.g., slips, trips, and other perturbations) must be more relevant to fall risk assessment than quiet stance.

Furthermore, Maki included both quiet stance and dynamic postural control measures and described potential methodological problems with dynamic tasks that yielded wide inter-participant variability in the performance of anticipatory tasks and a lack of real-world transient responses [1]. These limitations provide potential explanations for why quiet stance metrics were found to be the best predictors. Another advantage of using quiet stance sway metrics, as opposed to dynamic ones, is the suitability for use in nearly all clinical settings given the minimal space, equipment, and specialized expertise requirements. To replicate the categorical test of Agrawal one requires only a foam pad and a wristwatch. Quantifying sway, which we recommend because it is objective and also likely to yield higher sensitivity than a categorical assay, requires only the addition of either a force plate or Inertial Measurement Units (IMUs), without needing any additional specialized skills to score the test reliably. Nonetheless, while data clearly show that sway during quiet stance can be used to quantify fall risk, we also suggest that additional studies should be conducted to determine the relationship between vestibular thresholds and alternative, dynamic postural control measures in side-by-side comparisons with quiet stance metrics.

## DISCUSSION

The identified link between vestibular noise, assayed using vestibular thresholds, and quiet stance balance performance provides insight into one potential factor contributing to fall risk among otherwise healthy older adults. While correlation alone cannot prove causation, the identified robust correlation between roll tilt thresholds [25] and balance findings [22] alongside a robust correlation between the exact same balance findings and falls [12] suggest a causative chain. Specifically, we posit the following causal chain: (a) increased “noise” in vestibular feedback increases sway, (b) excessive sway leads to imbalance, and (c) imbalance contributes to falls. Furthermore, since the correlations shown both by Karmali, et al. [21], as well as Karmali, et al. [19] were made in healthy, asymptomatic populations, these results implicate vestibular noise as a potential cause of subclinical balance declines. As such, these data suggest that quiet stance balance screening could be used early to identify individuals with subclinical balance declines and/or to quantify fall risk. We suggest that when such a balance screening indicates increased fall risk, a follow-up test of vestibular thresholds could then identify (1) whether the subclinical balance impairment was due to vestibular noise and (2) which vestibular modality (e.g., roll tilt, lateral translation) is responsible for the observed balance impairment.

## CONCLUSIONS

We emphasize three additional points for consideration:

- Once balance screening has identified those at risk of falling, effective fall prevention will likely benefit from the inclusion of a detailed and biofidelic set of tests that quantify the integrity of multiple elements relevant to balance control (vestibular, vision, proprioception, strength, cognition, etc.).
- As additional data accumulate, dynamic balance tests that overcome previous methodological limitations may eventually replace the quiet stance balance screening if substantially stronger links to falls are measured in side-by-side comparisons.
- Use of such balance and vestibular screening tests could provide data to guide the use of therapeutic interventions designed specifically to prevent the onset of falls by improving the use of vestibular cues for postural control. Such methods may include rehabilitation focused on improving tilt and/or lateral translation sensation or the use of prosthetic devices to restore the perception of tilt and/or lateral translation cues.

## References

[1] Maki BE, Holliday PJ, Topper AK. A prospective study of postural balance and risk of falling in an ambulatory and independent elderly population. J Gerontol. 1994;49(2):M72–M84. 8126355 10.1093/geronj/49.2.m72

[2] Horak FB, Shupert CL, Mirka A. Components of postural dyscontrol in the elderly: A review. Neurobiol Aging. 1989;10(6):727–738. 2697808 10.1016/0197-4580(89)90010-9

[3] Wagner AR, Akinsola O, Chaudhari AM, Bigelow KE, Merfeld DM. Measuring vestibular contributions to age-related balance impairment: A review. Front Neurol. 2021;12:635305. 33633678 10.3389/fneur.2021.635305 PMC7900546

[4] Stevens JA, Mahoney JE, Ehrenreich H. Circumstances and outcomes of falls among high risk community-dwelling older adults. Inj Epidemiol. 2014;1(1):1–9. 26744637 10.1186/2197-1714-1-5 PMC4700929

[5] Houry D, Florence C, Baldwin G, Stevens J, McClure R. The CDC injury center’s response to the growing public health problem of falls among older adults. Am J Lifestyle Med. 2016;10(1):74–77. 10.1177/1559827615600137 PMC4681302 26688674

[6] Bergen G, Stevens MR, Burns ER. Falls and fall injuries among adults aged≥ 65 years-United States, 2014. Morb Mortal Wkly Rep. 2016;65(37):993–998. 10.15585/mmwr.mm6537a2 27656914

[7] Carter ND, Khan KM, Mallinson A, Janssen PA, Heinonen A, Petit MA, Knee extension strength is a significant determinant of static and dynamic balance as well as quality of life in older community-dwelling women with osteoporosis. Gerontology. 2002;48(6):360–368. 12393951 10.1159/000065504

[8] Anson E, Bigelow RT, Swenor B, Deshpande N, Studenski S, Jeka JJ, Loss of peripheral sensory function explains much of the increase in postural sway in healthy older adults. Front. Aging Neurosci. 2017;9:202. 28676758 10.3389/fnagi.2017.00202 PMC5476729

[9] Salonen L, Kivelä SL. Eye diseases and impaired vision as possible risk factors for recurrent falls in the aged: A systematic review. Curr Gerontol Geriatr Res. 2012;271481. 22927843 10.1155/2012/271481 PMC3426172

[10] Agrawal Y, Merfeld DM, Horak FB, Redfern MS, Manor B, Westlake KP, Aging, vestibular function, and balance: Proceedings of a national institute on aging/National institute on deafness and other communication disorders workshop. J Gerontol A Biol. 2020;75(12):2471–2480. 10.1093/gerona/glaa097 PMC7662183 32617555

[11] Agrawal Y, Smith P, Merfeld D. Dizziness, Imbalance and Age-Related Vestibular Loss. In: The Senses a Comprehensive Reference. 2021;6:567–580.

[12] Agrawal Y, Carey JP, Santina DCC, Schubert MC, Minor LB. Disorders of balance and vestibular function in US adults: Data from the national health and nutrition examination survey, 2001-2004.Arch Intern Med. 2009;169(10):938–944. 19468085 10.1001/archinternmed.2009.66

[13] Horak FB. Clinical measurement of postural control in adults. Phys Ther. 1987;67(12):1881–1885. 3685116 10.1093/ptj/67.12.1881

[14] Peterka RJ. Sensory integration for human balance control. In: Handbook of Clinical Neurology. 2018;159:27–42. 30482320 10.1016/B978-0-444-63916-5.00002-1

[15] Horak F, Nashner L, Diener H. Postural strategies associated with somatosensory and vestibular loss. Exp Brain Res. 1990;82(1):167–177. 2257901 10.1007/BF00230848

[16] Diener HC, Dichgans J. On the role of vestibular, visual and somatosensory information for dynamic postural control in humans. Prog Brain Res. 1988;76:253–262. 3064150 10.1016/s0079-6123(08)64512-4

[17] Angelaki DE, Cullen KE. Vestibular System: The many facets of a multimodal sense. Annu Rev Neurosci. 2008;31(1):125–150. 18338968 10.1146/annurev.neuro.31.060407.125555

[18] Rey BMC, Clark TK, Wang W, Leeder T, Bian Y, Merfeld DM. Vestibular perceptual thresholds increase above the age of 40. Front Neurol. 2016;7. 27752252 10.3389/fneur.2016.00162 PMC5046616

[19] Karmali F, Rey BMC, Clark TK, Wang W, Merfeld DM. Multivariate analyses of balance test performance, vestibular thresholds, and age. Front Neurol. 2017;8:578. 29167656 10.3389/fneur.2017.00578 PMC5682300

[20] Beylergil SB, Karmali F, Wang W, Rey BMC, Merfeld DM. Vestibular roll tilt thresholds partially mediate age-related effects on balance. In: Progress in Brain Research. 2019;248:249–267. 31239136 10.1016/bs.pbr.2019.04.019

[21] Wagner AR, Kobel MJ, Merfeld DM. Impact of canal-otolith integration on postural control. Front Integr Neurosci. 2021. 10.3389/fnint.2021.773008 PMC8713561 34970126

[22] Karmali F, Goodworth AD, Valko Y, Leeder T, Peterka RJ, Merfeld DM. The role of vestibular cues in postural sway. J Neurophysiol. 2021;125(2):672–686. 33502934 10.1152/jn.00168.2020 PMC7948142

[23] Thapa PB, Gideon P, Brockman KG, Fought RL, Ray WA. Clinical and biomechanical measures of balance as fall predictors in ambulatory nursing home residents. J Gerontol A Biol Sci Med Sci. 1996;51(5):M239–M246. 8808996 10.1093/gerona/51a.5.m239

[24] Stel VS, Pluijm SMF, Deeg DJH, Smit JH, Bouter LM, Lips P. A classification tree for predicting recurrent falling in community-dwelling older persons. J Am Geriatr Soc. 2003;51(10):1356–1364. 14511154 10.1046/j.1532-5415.2003.51452.x

[25] Klaus MP, Schöne CG, Hartmann M, Merfeld DM, Schubert MC, Mast FW. Roll tilt self-motion direction discrimination training: First evidence for perceptual learning. Atten Percept Psychophys. 2020;82:1987–1999. 31898068 10.3758/s13414-019-01967-2 PMC7297830

